# CLARITY – ChiLdhood Arthritis Risk factor Identification sTudY

**DOI:** 10.1186/1546-0096-10-37

**Published:** 2012-11-15

**Authors:** Justine A Ellis, Anne-Louise Ponsonby, Angela Pezic, Raul A Chavez, Roger C Allen, Jonathan D Akikusa, Jane E Munro

**Affiliations:** 1 Genes, Environment and Complex Disease, Murdoch Childrens Research Institute, Parkville, VIC, Australia; 2 Environmental and Genetic Epidemiology Research, Murdoch Childrens Research Institute, Parkville, VIC, Australia; 3 Arthritis & Rheumatology, Murdoch Childrens Research Institute, Parkville, VIC, Australia; 4 Paediatric Rheumatology Unit, Department of General Medicine, The Royal Children’s Hospital, Parkville, VIC, Australia; 5 Department of Physiology, The University of Melbourne, Parkville, VIC, Australia

**Keywords:** Juvenile idiopathic arthritis, Epidemiology, Demographics, Early life, Risk factors

## Abstract

**Background:**

The aetiology of juvenile idiopathic arthritis (JIA) is largely unknown. We have established a JIA biobank in Melbourne, Australia called CLARITY – ChiLdhood Arthritis Risk factor Identification sTudY, with the broad aim of identifying genomic and environmental disease risk factors. We present here study protocols, and a comparison of socio-demographic, pregnancy, birth and early life characteristics of cases and controls collected over the first 3 years of the study.

**Methods:**

Cases are children aged ≤18 years with a diagnosis of JIA by 16 years. Controls are healthy children aged ≤18 years, born in the state of Victoria, undergoing a minor elective surgical procedure. Participant families provide clinical, epidemiological and environmental data via questionnaire, and a blood sample is collected.

**Results:**

Clinical characteristics of cases (n = 262) are similar to those previously reported. Demographically, cases were from families of higher socio-economic status. After taking this into account, the residual pregnancy and perinatal profiles of cases were similar to control children. No case-control differences in breastfeeding commencement or duration were detected, nor was there evidence of increased case exposure to tobacco smoke in utero. At interview, cases were less likely to be exposed to active parental smoking, but disease-related changes to parent behaviour may partly underlie this.

**Conclusions:**

We show that, after taking into account socio-economic status, CLARITY cases and controls are well matched on basic epidemiological characteristics. CLARITY represents a new study platform with which to generate new knowledge as to the environmental and biological risk factors for JIA.

## Background

JIA is defined as a chronic autoimmune inflammatory arthritis of largely unknown aetiology that begins before 16 years of age [1]. It is characterised by joint swelling, pain or tenderness, and movement limitation not due to a primary mechanical disorder, that persists for at least 6 weeks. Cases are classified into seven subtypes, based on the number of joints affected and other disease features, using the International League of Associations for Rheumatology (ILAR) classification system. Current treatments are aimed at reducing pain and inflammation, but they are largely not based on known aetiology and are thus not optimally effective [1].

The prevalence of JIA has been estimated to fall between 0.07 and 4 per thousand Caucasian children [2]. JIA can have significant impact in terms of decreased quality of life, physical function, and development [3].

JIA is typical of autoimmune disease, in that it is considered a complex disease, with susceptibility dependent on a complex interplay between inherited genetic variants [4], and life course exposure to adverse environments [5]. Amongst the relatively small number of genetic risk variants robustly identified are those in the Human Leukocyte Antigen (HLA) region, and in the shared autoimmunity gene *PTPN22*[6]. Promisingly, a number of new gene loci have recently been reported, including *VTCN1*[7], *AFF3*[8], *IL2RA*[9], *PTPN2*[10], and C3orf1/CD80 [11] however these loci generally await further confirmation by independent studies. As we recently reviewed, less is known about the environmental factors that contribute to JIA risk [5]. Recent work identifying factors such as UVR exposure and Vitamin D, and exposure to microbes during early life (the hygiene hypothesis) and at disease onset, as important in developing other autoimmune diseases, provide hypothesis-generating clues for future research [5]. However, there is a paucity of study platforms available with which to examine environmental factors, and their interactions with gene variants.

Here, we provide detail of CLARITY, the ChiLdhood Arthritis Risk factor Identification sTudY, established in Melbourne, Australia, to address these knowledge gaps. We present study design and data collection methodologies, along with recruitment, biospecimen and environmental data collection rates, and clinical, demographic, prenatal, birth and lifestyle characteristics of the first 314 cases and 481 controls recruited to CLARITY, from study commencement in February 2008 until December 2010.

## Methods

### Case recruitment

All CLARITY protocols are approved by the Human Research Ethics Committee of the Royal Children’s Hospital, Melbourne Australia. All participants provided written consent.

Cases are recruited during a public or private clinic visit to the Royal Children’s Hospital (RCH), Victoria, Australia. The RCH is located in central Melbourne, and is the major paediatric tertiary referral hospital in the state. An estimated 80% of all Victorian JIA cases attend the RCH paediatric rheumatology clinic serviced by three rheumatologists.

Case inclusion criteria are that the child is aged between 0-18 years at interview, with diagnosis of JIA by a paediatric rheumatologist before the age of 16 years. Exclusion criteria are the presence of major congenital abnormalities, or illness that would forgo school attendance in the one year prior to recruitment. Incident cases are defined as children recruited within six months of diagnosis. Prevalent cases are defined as those children diagnosed more than 6 months before recruitment, and since 1997. Cases were diagnosed with JIA using the ILAR criteria [12]. Case families complete a questionnaire gathering information about the child’s birth; the first years of life; the household and family; skin type, sun exposure and activities; sleep habits; health problems; teeth; atopic disease; and family illness history. Additionally, case families complete a questionnaire gathering information about the parents, including socio-demographic measures, ancestry, lifestyle during the child’s pregnancy (e.g. smoking, alcohol use), contact with animals and sick people, illnesses, and sun exposure; skin type; current smoking and smoking indoors near the child. Cases provide a 9 ml peripheral blood sample.

### Control recruitment

In recognition of the difficulties in obtaining a blood specimen from population/community-based child controls, controls are recruited through the Royal Children’s Hospital Day Surgery Unit (RCH DSU).

Families are invited to participate if their child is aged between 0-18, a patient of the RCH DSU for the purposes of elective surgery, and was born in the state of Victoria. This criterion allows the representativeness of the hospital based control group to the Victorian paediatric population to be eventually assessed via comparison of collected birth summary data to that collected within the Victorian Perinatal Data Collection Unit (VPDCU) [13]. Differences in demographic characteristics of controls compared to the Victorian population as a whole can then be accounted for during data analyses using back-weighting adjustments, as we have used in previous work [14]. Cases were not similarly restricted in recognition of their more limited availability, and the utility of all cases for genomic analyses regardless of place of birth. Control families are excluded if the child has major congenital abnormalities, or illness that would forgo school attendance in the year prior to recruitment. Control families complete questionnaires covering the same child and parent items as for the cases. Control children provide a 9 ml peripheral blood sample, collected prior to anaesthesia.

### Questionnaire data

In most instances, questionnaires are completed on the day of recruitment in the presence of a research nurse. Occasionally, due to time constraints, the questionnaire is completed at home. A research nurse is made available to participants by telephone to answer queries. Data is scanned directly into a comma separated file using the Teleform^©^ system.

### Biobanking procedures

The peripheral blood sample is collected into EDTA and immediately delivered to the onsite biobanking facility. Plasma is removed within 2 hours and stored at -80°C. Within 24 hours, peripheral blood mononuclear cells (PBMCs) are isolated using a standard ficoll procedure, chilled slowly to -80°C, then transferred to vapour-phase liquid nitrogen storage to maintain cell viability. The remaining white blood cells (mainly consisting of granulocytes) are also isolated for extraction of genomic DNA.

Consent is also sought from both cases and controls to access Newborn Screening Cards – newborn (within 72 hours) blood spotted to card for over 99% of Victorian births for screening of treatable disease such as phenylketonuria (PKU). These cards are retained indefinitely by Genetic Health Services Victoria (housed at RCH). Newly developed methods allow the use of these blood spots for analysis of potential disease biomarkers, such as vitamin D [15], and DNA methylation [16].

### Statistical analyses

In this paper we present data collected to CLARITY from February 2008 (study commencement) until December 2010, for a comparison of collected data pertaining to socio-demographics, lifestyle and birth between cases and controls. Limiting data to participants recruited by December 2010 provided a 12 month window to clarify clinical diagnosis of JIA, prior to finalisation of data for analysis.

Logistic regression was used to examine case-control differences. Analyses were carried out on the full dataset, and on the dataset restricted to cases (and controls) born in Victoria. Unadjusted odds ratios (UOR) were calculated. ORs for Victorian born cases vs controls were then adjusted (AOR) for maternal socio-economic status using SEIFA score (Socio-Economic Indexes for Areas, a ranking of geographic postal-code areas in terms of socio-economic characteristics, Australian Bureau of Statistics) [17], child age, sex and Caucasian ancestry (yes/no), and maternal age at the child’s birth. Paternal SEFIA and age at child’s birth were not additionally adjusted for since maternal and paternal measures were highly correlated (SEIFA score r = 0.90, p < 0.0001; age at child’s birth r = 0.70, p < 0.0001). Further adjustment for parental age at interview did not materially alter the ORs. A similar approach was taken for the comparison of older (> 6 years) to younger (≤ 6 years) diagnosed case data, except that these analyses were carried out on all cases, and were not adjusted for child age. A p < 0.05 was considered significant. All analyses were performed using Stata v11 (StataCorp, College Station, TX).

## Results

### Recruitment and collection statistics

Of the cases, 95% (314/330) of families that were deemed eligible by our criteria took up an invitation to participate. For controls identified within the RCH DSU, we have achieved an overall recruitment rate of 89% (481/540) by face-to-face recruitment of families following child admission procedures and prior to entering theatre.

The questionnaire was completed (or partially completed) by 87% (272/314) of cases, and 95% (458/481) of controls. Plasma was collected for 91% (286/314) of cases and 98% (471/481) of controls, and PBMCs were collected for 86% (270/314) of cases and 90% (433/481) of controls.

### Completeness of data

Eight cases were excluded from analysis because, although their diagnosis at recruitment was JIA, this diagnosis had changed by commencement of data analysis. Data from two cases who withdrew from the study was also excluded. Examination of Table 1 demonstrates that data is not complete for all variables for the remaining 262 cases and 458 controls who completed/partially completed questionnaires. In general, the proportion of missing data is low. However, several changes to the questionnaires occurred following study piloting. Variables added post-study piloting include SEIFA score, mode of delivery, and indoor parental smoking.

**Table 1:** Comparison of basic data collected in CLARITY cases and controls

	**All cases**		**Victorian-born cases**		**Victorian-born (all) controls**	
	**n†**		**n†**		**n†**	
***Basic characteristics***						
Child mean age at interview	262	9.5 (SD: 4.5)*	229	9.4 (SD: 4.6)*	458	7.1 (SD 4.2)
Child female	262	67.2%*	229	68.1%*	458	39.7%
Child has four Caucasian grandparents	217	87.1%*	190	90.0%*	378	79.1%
Mother’s age at interview	256	40.3 (SD: 6.0)*	224	40.5 (SD: 5.8)*	449	36.9 (SD: 6.4)
Father’s age at interview	243	43.0 (SD: 6.6)*	213	43.2 (6.5)*	398	39.4 (SD: 6.8)
***Socio***-***demographic data***						
Mother’s SEIFA^§^ score	90	1026.7 (SD: 62.9)*	74	1030.9 (SD: 62.6)*	305	1003.7 (SD: 70.4)
Father’s SEIFA^§^ score	81	1026.1 (SD: 64.3)*	67	1033.6 (SD: 61.7)*	245	1006.5 (SD: 71.7)
Mother’s education						
Completed year 12	257	66.2%	224	68.8%	449	71.9%
Completed postgraduate degree	202	10.4%	173	10.4%	344	10.8%
Father’s education						
Completed year 12	244	57.4%	215	57.7%	399	61.4%
Completed postgraduate degree	180	12.8%	157	12.7%	275	9.8%
Mother’s marital status at interview	256		223		450	
married		79.3%*		80.7%*		70.9%
divorced		7.0%		7.2%		6.2%
separated		5.1%		4.9%		6.2%
widowed		0.8%		0.5%		1.3%
de facto		2.0%		1.8%		4.9%
never married		5.9%		4.9%		10.2%
Father’s marital status at interview	246		216		401	
married		84.2%*		84.3%*		75.8%
divorced		5.7%		5.6%		4.2%
separated		3.7%		3.7%		5.7%
widowed		0.8%		0.9%		0%
de facto		1.2%		1.4%		3.7%
never married		4.1%		3.7%		9.2%
don’t know		0.4%		0.5%		1.3%
Mother hours in paid work (mean)	220	23.0 (SD: 20.0)	193	22.9 (SD: 19.9)	340	20.2 (SD: 19.1)
Father hours in paid work (mean)	216	43.0 (SD: 15.1)	190	43.3 (SD: 15.1)	312	41.5 (SD: 16.5)
Mother cigarettes/day	255		222		451	
none		80.0%*		79.7%*		72.7%
1-10/day		9.8%		9.9%		14.9%
11-20/day		8.2%		8.6%		10.2%
21-40/day		2.0%		1.8%		2.0%
41+/day		0%		0%		0.2%
Father cigarettes/day	242		213		397	
none		78.1%		79.3%*		71.5%
1-10/day		7.9%		8.0%		10.8%
11-20/day		10.3%		9.4%		10.8%
21-40/day		2.9%		2.8%		6.3%
41+/day		0.8%		0.5%		0.5%
Mother alcoholic drinks/wk mean (SD)	249	2.2 (SD: 3.8)	216	2.4 (SD: 4.0)	435	1.9 (SD: 3.0)
Father alcoholic drinks/wk mean (SD)	232	4.9 (SD: 6.6)	204	4.8 (SD: 6.6)	384	5.1 (SD: 7.9)
***Pregnancy *&*Birth***						
Mother’s age at child’s birth (mean, yrs)	256	30.2 (SD: 4.9)*	224	30.6 (SD: 4.8)*	449	29.3 (SD: 5.3)
Father’s age at child’s birth (mean, yrs)	243	33.0 (SD: 5.7)*	213	33.4 (SD: 5.7)*	398	32.0 (SD: 6.1)
Planned pregnancy	262	79.8%	229	81.2%	456	77.6%
planned natural		73.7		75.6%		72.8%
planned assisted		6.1%		5.7%		4.8%
unplanned		20.2%		18.8%		22.4%
Child adopted	262	0%	229	0%	455	0.7%
Mother smoked during this pregnancy	256		223		448	
Nil		84.0%*		83.9%*		74.6%
Less than daily		5.9%		6.3%		10.0%
1-10/day		6.3%		6.7%		9.4%
11-20/day		3.5%		3.2%		4.7%
21-40/day		0.4%		0%		1.3%
41+/day		0%				0%
Father smoked during this pregnancy	235		207		385	
Nil		77.0%*		78.3%*		68.8%
Less than daily		3.0%		2.9%		4.9%
1-10/day		5.1%		5.3%		9.6%
11-20/day		11.1%		10.1%		11.4%
21-40/day		3.4%		3.4%		4.9%
41+/day		0.4%		0%		0.3%
Mother major illness during pregnancy	261	26.1%	228	25.0%	452	23.0%
Mother meds/supps during pregnancy						
multivitamins	245	24.9%	215	25.1%	409	30.8%
folate	246	61.4%	216	60.7%	409	64.8%
calcium	246	10.2%	216	9.7%	409	14.9%
iron	246	35.4%	216	35.6%	409	40.3%
vitamin D	246	3.3%*	216	3.7%	409	7.8%
fish oil	246	2.9%	216	2.8%	409	6.4%
antibiotics	246	3.7%	216	3.7%	409	3.7%
other	244	11.5%*	214	11.2%	409	6.4%
Mother any alcohol during pregnancy	255	18.1%	222	19.4%*	448	18.3%
Mother any coffee during pregnancy	253	56.1%*	220	57.7%*	448	54.7%
Child’s gestation (mean, weeks)	211	39.3 (SD: 1.8)	186	39.3 (SD: 1.8)	389	39.0 (SD: 2.3)
Child mode of delivery	90		71		210	
normal vaginal		63.3%		63.4%		60.0%
assisted vaginal		12.2%		12.7%		11.9%
caesarian		23.3%		22.5%		27.1%
other		1.1%		1.4%		1.0%
Child birthweight (mean, g)	225	3354.0 (SD: 613.8)	200	3357.2 (SD: 581.5)	344	3363.0 (SD: 676.9)
Child birthlength (mean, cm)	194	50.2 (SD: 3.4)	174	50.1 (SD: 3.4)	268	50.4 (SD: 3.7)
Child head circumference (mean, cm)	161	34.8 (SD: 3.0)	149	34.8 (SD: 3.1)	176	34.6 (SD: 3.1)
Child in multiple birth	253	3.6%	221	4.1%	448	3.1%
Child birth order (all live-born sibs) mean	262	1.90 (SD:1.02)	229	1.93 (SD: 1.05)	456	1.85 (SD: 1.03)
***Early Life***						
Child breastfeeding						
any	260	85.8%	228	86.4%	454	81.3%
if any, age started (wks) (mean, SD)	217	0.3 (SD 1.6)	191	0.3 (SD 1.7)	360	0.9 (SD 5.0)
if any, weeks breastfed (mean, SD)	214	36.5 (SD 29.9)	188	36.5 (SD 30.4)	357	37.6 (SD 34.4)
Child formula feeding						
age started (weeks)	194	18.2 (SD 19.9)	173	18.3 (SD 20.1)	348	16.4 (SD 16.5)
weeks formula fed	239	35.1 (SD 34.3)	210	35.9 (35.2)	433	37.2 (SD 37.1)
Child cow’s milk commence age (weeks)	221	58.8 (SD 17.6)	193	57.9 (SD 17.6)*	405	61.6 (SD 22.4)
Child solids commence age (weeks)	234	24.2 (SD 16.1)	210	23.8 (SD 16.4)	424	23.9 (SD 9.6)
Mother smokes indoors near child	241		211		216	
usually		0.8%		1.0%		1.4%
sometimes		3.7%		3.8%		6.9%
never		95.4%		95.3%		91.7%
Father smokes indoors near child	231		203		192	
usually		1.3%		1.0%		2.6%
sometimes		3.5%		3.5%		5.7%
never		95.2%		95.6%		91.7%

† Number of observations.

* p < 0.05 vs controls by unadjusted logistic regression. See Table 2 for adjusted data.

§ SEIFA = Socio-Economic Indexes for Areas, a measure of residential positional disadvantage based on the Australian Bureau of Statistics Census of Population and Housing. The higher the score, the lower the disadvantage.

### Case-control data comparisons

Table 1 displays characteristics of all cases, Victorian-born cases, and Victorian-born (all) controls. Table 2 summarises logistic regression analyses comparing Victorian-born cases and controls for selected variables, adjusting sequentially for mother’s SEIFA score, child measures (age, sex, ancestry), and mother’s age at the child’s birth. Below, we highlight some of the key findings from data presented in these tables.

**Table 2:** Unadjusted and adjusted logistic regression odds ratios (UOR/AOR) between Victorian-born cases and controls for selected phenotypic ‘exposure’ variables

	**UOR (95% CI)**	**AOR (95% CI)**		
		**Mother’s SEIFA score**	**Child measures†**	**Mother’s age at child’s birth**
Mother’s SEIFA score*	1.01 (1.00, 1.01), p = 0.003	**	1.01 (1.00, 1.01), p = 0.004	**1.01 (1.00, 1.01), p = 0.011**
**Mother completed Yr12**	0.86 (0.61, 1.22), p = 0.39	1.02 (0.55, 1.88), p = 0.95	2.95 (1.30, 6.68), p = 0.009	**2.72 (1.19, 6.20) p = 0.017**
Father completed Yr12	0.86 (0.61, 1.20), p = 0.37	0.64 (0.37, 1.11), p = 0.11	1.05 (0.55, 1.98), p = 0.89	0.98 (0.51, 1.86), p = 0.94
**Mother married at interview**	1.71 (1.15, 2.52), p = 0.007	1.82 (0.96, 3.45), p = 0.065	2.94 (1.32, 6.57), p = 0.009	**2.62 (1.16, 5.92), p = 0.020**
**Father married at interview**	1.67 (1.08, 2.59), p = 0.022	2.11 (1.02, 4.39), p = 0.044	2.78 (1.20, 6.43), p = 0.017	**2.48 (1.06, 5.80), p = 0.036**
Mother hours in paid work*	1.01 (1.00, 1.02), p = 0.12	0.99 (0.98, 1.01), p = 0.40	0.98 (0.96, 1.00), p = 0.078	**0.98 (0.96, 1.00), p = 0.037**
Father hours in paid work*	1.01 (1.00, 1.02), p = 0.23	1.01 (0.99, 1.03), p = 0.50	1.02 (0.99, 1.04), p = 0.17	1.02 (0.99, 1.04), p = 0.16
**Mother currently smokes**	0.68 (0.46, 1.00), p = 0.049	0.45 (0.22, 0.92), p = 0.029	0.25 (0.095, 0.63), p = 0.003	**0.27 (0.11, 0.71), p = 0.008**
Father currently smokes	0.65 (0.44, 0.97), p = 0.036	0.68 (0.36, 1.30), p = 0.25	0.69 (0.34, 1.41), p = 0.31	0.80 (0.39, 1.64), p = 0.54
Mother’s age at child’s birth*	1.05 (1.02, 1.08), p = 0.002	1.07 (1.01, 1.12), p = 0.016	**1.08 (1.02, 1.15), p = 0.011**	**
Father’s age at child’s birth*	1.04 (1.01, 1.07), p = 0.009	1.04 (1.00, 1.09), p = 0.067	**1.06 (1.01, 1.11), p = 0.025**	**
Mother smoked during child’s pregnancy (any)	0.56 (0.37, 0.85), p = 0.007	0.77 (0.41, 1.45), p = 0.43	0.52 (0.24, 1.12), p = 0.094	0.61 (0.28, 1.33), p = 0.21
**Father smoked during child’s pregnancy (any)**	0.61 (0.41, 0.91), p = 0.015	0.52 (0.26, 0.97), p = 0.039	0.42 (0.20, 0.89), p = 0.024	**0.46 (0.21, 0.99), p = 0.047**
Mother took Vitamin D during child’s pregnancy	0.45 (0.21, 1.00), p = 0.05	0.16 (0.02, 1.21), p = 0.075	0.21 (0.03, 1.69), p = 0.14	0.26 (0.03, 2.10), p = 0.21
Mother took fish oil during child’s pregnancy	0.42 (0.17, 1.04), p = 0.06	0.45 (0.10, 1.99), p = 0.29	0.71 (0.15, 3.36), p = 0.66	0.70 (0.15, 3.33), p = 0.65
Mother drank alcohol during child’s pregnancy (any)	1.07 (0.71, 1/62), p = 0.74	0.72 (0.35, 1.48), p = 0.37	1.07 (0.50, 2.35), p = 0.85	0.97 (0.44, 2.16), p = 0.94
Mother drank coffee during child’s pregnancy (any)	1.13 (0.82, 1.57), p = 0.46	0.85 (0.51, 1.42), p = 0.53	0.67 (0.37, 1.21), p = 0.19	0.66 (0.36, 1.22), p = 0.19
Child was breastfed (any)	1.46 (0.94, 2.29), p = 0.094	0.88 (0.44, 1.73), p = 0.70	0.90 (0.41, 1.97), p = 0.79	0.86 (0.39, 1.89), p = 0.71
Length of breastfeeding (if any, weeks)*	1.00 (0.99, 1.00), p = 0.70	1.00 (1.00, 1.01), p = 0.19	1.01 (1.00, 1.01), p = 0.25	1.00 (0.99, 1.01), p = 0.72
Age at commencement of cow’s milk (weeks)*	0.99 (0.98, 1.00), p = 0.043	1.00 (0.99, 1.01), p = 0.88	0.99 (0.98, 1.01), p = 0.45	0.99 (0.98, 1.01), p = 0.30
Mother smokes indoors near child (any)	0.55 (0.25, 1.21), p = 0.14	0.49 (0.21, 1.10), p = 0.082‡	0.32 (0.10, 1.01), p = 0.052	0.33 (0.10, 1.06), p = 0.063
**Father smokes indoors near child (any)**	0.51 (0.22, 1.18), p = 0.12	0.52 (0.22, 1.23), p = 0.14‡	0.22 (0.07, 0.67), p = 0.008	**0.26 (0.08, 0.81), p = 0.020**

Covariates were added to the logistic regression model sequentially from left to right.

Phenotypic variables that remained significantly different between cases and controls following full covariate adjustment are highlighted in **bold**.

†Child measures = age, sex, Caucasian ancestry (y/n).

*β coefficients reported for continuous exposures.

** Not adjusted for since same as, or highly significantly correlated with, the exposure variable.

‡ Mother’s completion of high school used in the model as a proxy for mother’s SEIFA score (correlation: r = 0.24, p < 0.0001) due to insufficient data for SEIFA.

#### Socio-demographic data

Based on SEIFA score, mothers of Victorian-born cases were generally more advantaged than mothers of controls. Data for fathers was similar to that for mothers. More mothers of Victorian-born cases were married at the time of interview. There was evidence that mothers of cases were more highly educated, but worked fewer paid hours, than mothers of controls, following covariate adjustments.

#### Parental lifestyle

Fewer mothers and fathers of Victorian-born cases reported any smoking at the time of interview; this difference remained significant following regression adjustments for the mother, but not the father. The number of reported alcoholic drinks per week at the time of interview was not different between cases and controls for either mothers or fathers.

#### Pregnancy and birth

All data collected on pregnancy and birth pertains to the pregnancy and birth of the child recruited to the CLARITY study.

Both mothers and fathers of Victorian-born cases were significantly older at the time of the child’s birth. Significantly fewer mothers and fathers of Victorian-born cases reported any smoking during the pregnancy, this association persisted for fathers, but not for mothers, following covariate adjustments.

There was some evidence of association of JIA with nutritional supplementation during pregnancy. Use of vitamin D and fish oil during pregnancy was lower in case mothers; however, these associations were not significant for Victorian-born participants following covariate adjustments.

No differences between cases and controls were observed for gestation length or mode of delivery (including caesarean vs non-caesarean birth). Similarly, birth weight, birth length, head circumference and frequency of a child born within a multiple birth were not different between the groups. In relation to other siblings, the child’s birth order was also not different between cases and controls.

#### Early life

Breastfeeding was commenced in 86% of Victorian-born cases, and 81% of controls; these differences were not significant. Amongst those who were breastfed, there were no significant differences between Victorian-born case and control children for breastfeeding duration. Amongst those who were formula fed, there were also no significant differences in age at commencement. A significant difference was detected for age at cow’s milk introduction; on average, Victorian-born cases commenced cow’s milk at a younger age than controls. However, this association did not persist following covariate adjustments. No differences in the age at commencement of solids were detected.

The frequency of both mothers and fathers who reported any smoking indoors near the child was lower in Victorian-born cases compared to controls. This difference was significant following covariate adjustments for fathers, but not for mothers. For these analyses, completion of high school by the mother was used in place of SEIFA score as a measure of socioeconomic status, since there was insufficient data for SEIFA score in the model, and the two socioeconomic covariates were significantly correlated (p < 0.0001).

### Comparison of data between younger- and older-diagnosed cases

We also considered whether differences might be evident between cases diagnosed at 6 years of age or younger (younger diagnosed cases) and cases diagnosed after 6 years of age (older diagnosed cases). We chose a cut-point of 6 years for two reasons. Firstly, the median age at diagnosis was 6.4 years, and therefore the cases were approximately evenly distributed using this cut-point. Secondly, there is evidence to suggest that the underlying biological characteristics of JIA may be different between cases grouped in this way, even amongst cases of the same subtype [18,19].

Additional file 1: Table S1 presents full data on characteristics of younger and older-diagnosed cases. Only a few statistically significant differences were identified when these case group definitions were compared. Of note, the percentage of females was higher in younger diagnosed cases; this likely reflects the fact that common subtypes such as oligoarticular JIA that are more commonly diagnosed at a younger age are also more often diagnosed in females [20]. We also noted a difference in the age of both mothers and fathers at the time of birth of the case child. Mothers of younger diagnosed cases were older (β = 1.1; 95% CI 1.0, 1.1; p = 0.015) as were fathers of younger diagnosed cases (β = 1.1; 95% CI 1.0, 1.1; p = 0.012) at the time of the case birth. However, these associations did not persist following adjustment for SEIFA score, and child sex and ancestry. Younger diagnosed case children were also less likely to have been born as part of a multiple birth, but not significantly so following covariate adjustments. Younger-diagnosed children were less likely to have a mother or father who smokes indoors near the child. These differences were not significant following adjustment for mother’s completion of high school (as a proxy for SEIFA score), child sex, and maternal age at birth. Insufficient data was available to add ancestry to the model. No other statistically significant differences between the younger and older case groups were observed.

## Discussion

The CLARITY JIA Biobank project was established in response to the paucity of data concerning both the genetic and environmental risk factors that contribute to JIA disease risk. Since its inception, the study team have achieved high recruitment rates. For cases, this demonstrates the high motivation of case families to participate in research regarding their disease. For controls in whom there is generally no such motivation to participate, we have achieved similarly high recruitment rates. Recruitment efforts have resulted in near-complete collection of questionnaire data and biospecimens (including carefully stored plasma and viable PBMCs) across all participants.

The data presented in this paper represents participants recruited between study commencement and December 2010. The 12 month lag-time prior to finalisation of data allowed for the confirmation of diagnosis of JIA. Eight cases were excluded from analysis (and two cases withdrew) during this 12 month window due to a change in diagnosis from JIA to ‘other arthritis’ (n = 7) or ‘ankylosing spondylitis’ (n = 1), and thus the imposed diagnosis window allowed a more accurate summation of data in confirmed JIA cases.

Collection of healthy paediatric control samples, especially where a blood sample is required, is a difficult task. Ideally, the control participants would be carefully sampled to accurately reflect the demographics of the entire Victorian population. However, in studies that require the collection of a biospecimen, particularly a blood sample, hospital-based controls are a necessary compromise between population-based sampling and achievement of sufficient recruitment rates. In our setting, we will have the ability to assess the robustness of full study findings to hospital control use through the collection of data on reason for minor surgery (e.g. infection related vs non-infection related admissions), and an ability to compare control demographic and birth data to that collected for all Victorian live births to the Victorian Perinatal Data Collection Unit [21].

Overall, these early case-control data comparisons demonstrate that the case and control groups are similar in many of their pregnancy, birth, and early life characteristics. Maternal SEIFA score proved to be an important covariate; with shifts in ORs and p values often evident upon adjustment for this variable. Some case-control differences were evident, even after adjustment for maternal SEIFA score, child age, sex and ancestry, and maternal age at the child’s birth. These included a higher level of education, but a lower number of hours in paid work for mothers of cases, possibly reflecting an increased parental care burden on mothers of children with chronic illness. A higher number of parents of cases were married at interview. Other characteristics were not different between cases and controls following covariate adjustments.

Smoking has been identified as a strong environmental risk factor for adult rheumatoid arthritis (RA) [22]; however our data shows no increase in the risk of JIA related to tobacco smoke exposure, either in utero, or during early life. At interview, cases were less likely to be exposed to active parental smoking, but disease-related changes to parent behaviour may partly underlie this.

Three small studies were published in the mid-1990’s that examined the impact of commencement and duration of breastfeeding on JIA disease risk. Each study carried significant limitations in terms of design and sample size (reviewed in [5]). However, two studies found that there was no difference in the commencement of breastfeeding [23,24], whilst one concluded that children with ‘juvenile rheumatoid arthritis’ were less likely to have been breastfed [25]. In relation to duration, one study found that longer duration of breastfeeding was protective against JIA [24], while one study found that duration was longer in children with polyarticular JIA compared to ‘pauciarticular’ (oligoarticular) JIA [23]. Our data showed a slightly higher rate of breastfeeding commencement in JIA children, although this difference was not significant. There were no differences in the duration of breastfeeding between cases and controls in our dataset. Overall, our data does not support a role for commencement or duration of breastfeeding in determining disease risk. There was some evidence for earlier introduction of cow’s milk in cases; earlier introduction of cow’s milk protein has been associated with other paediatric immune disorders [26]. However, the difference was not significant following adjustment for SEIFA score, suggesting this difference might be more related to socio-economic status.

There is a growing body of literature as to the role of vitamin D in autoimmune disease, including RA (reviewed in [5]). Interestingly, we found a trend towards a protective effect of the use of vitamin D and fish oil nutritional supplements during pregnancy, although this effect was not significant on adjustment for covariates. Additionally, the role of microbial exposures and infections in early life as potential environmental factors that protect against autoimmune disease (the hygiene hypothesis) [27] and/or act as disease triggers [28] is of interest. Caesarean delivery has been associated with increased risk of childhood immune disorders, and it has been proposed that this may be related to a lack of exposure to vaginal and intestinal flora during birth [29]. Our data shows no risk association with caesarean birth. However, a more complete examination of the role of microbial exposure on JIA risk is required to properly address such hypotheses.

## Conclusions

In summary, CLARITY is an internationally unique collection of clinical and environmental epidemiological data matched with biospecimens collected from children with JIA, and from healthy control children. Recruitment is ongoing. The data presented in this paper represents a series of cases and controls collected over the first 3 years of recruitment. Cases and controls were shown to be relatively comparable in terms of pregnancy, birth, and early life characteristics, and can be assessed for source population representativeness, providing real opportunities for novel risk factor identification in this understudied but burdensome childhood disease.

## Competing interests

The authors declare no competing interests.

## Authors' contributions

JE, JM and ALP conceived, designed and led the study. AP managed the study data and assisted JE with statistical analyses. RC assisted with the design of biobanking protocols and performed biobanking procedures. RA and JA assisted with case study design, and JM, RA and JA assisted with recruitment through their paediatric rheumatology clinics. JE wrote the manuscript, and all authors participated in drafting the manuscript to the final version.

## Supplementary Material

Additional file 1 **Table S1.** Comparison of basic data collected in CLARITY younger diagnosed and older diagnosed cases. Data not essential to the main message of the manuscript. Click here for file
