# Supplementary material for: CLARITY – ChiLdhood Arthritis Risk factor Identification sTudY
Source: Pediatr Rheumatol Online J. 2012 Nov 15;10:37. doi: 10.1186/1546-0096-10-37 (PMC3551677; doi:10.1186/1546-0096-10-37)
Supplement: Additional file 1 — Table S1. Comparison of basic data collected in CLARITY younger diagnosed and older diagnosed cases. Data not essential to the main message of the manuscript. [file 1546-0096-10-37-S1.docx]

**Supplementary Table 1:** Comparison of basic data collected in CLARITY younger diagnosed and older diagnosed cases.

|  | Younger diagnosed Cases  (≤ 6 years) | | Older diagnosed Cases  (> 6 years) | |
| --- | --- | --- | --- | --- |
|  | n† |  | n† |  |
|  |  |  |  |  |
| ***Basic characteristics*** |  |  |  |  |
|  |  |  |  |  |
| Child female | 118 | 73.7%* | 137 | 59.9% |
| Child has four Caucasian grandparents | 99 | 86.9% | 114 | 86.8% |
| Mother’s age at interview | 116 | 38.4 (SD: 5.8)* | 133 | 41.8 (SD: 5.7) |
| Father’s age at interview | 107 | 41.3 (SD: 6.4)* | 130 | 44.5 (6.4) |
|  |  |  |  |  |
| ***Socio-demographic data*** |  |  |  |  |
|  |  |  |  |  |
| Mother’s SEIFA^§^ score | 37 | 1027.0 (SD: 69.2) | 50 | 1024.6 (SD: 59.3) |
| Father’s SEIFA^§^ score | 32 | 1034.7 (SD: 59.7) | 47 | 1017.8 (SD: 67.2) |
| Mother’s education |  |  |  |  |
| Completed year 12 | 117 | 72.7% | 133 | 60.9% |
| Completed postgraduate degree | 85 | 8.2% | 112 | 11.6% |
| Father’s education |  |  |  |  |
| Completed year 12 | 110 | 59.1% | 129 | 55.0% |
| Completed postgraduate degree | 79 | 11.4% | 97 | 13.4% |
| Mother’s marital status at interview | 116 |  | 133 |  |
| married |  | 81.9% |  | 77.4% |
| divorced |  | 5.2% |  | 8.3% |
| separated |  | 4.3% |  | 6.0% |
| widowed |  | 0.9% |  | 0.8% |
| de facto |  | 0.9% |  | 3.0% |
| never married |  | 6.9% |  | 4.5% |
| Father’s marital status at interview | 110 |  | 131 |  |
| married |  | 89.1% |  | 80.2% |
| divorced |  | 3.6% |  | 7.6% |
| separated |  | 2.7% |  | 4.6% |
| widowed |  | 0.0% |  | 1.5% |
| de facto |  | 0.0% |  | 2.3% |
| never married |  | 4.6% |  | 3.1% |
| don’t know |  | 0.0% |  | 0.8% |
| Mother hours in paid work (mean) | 97 | 19.0 (SD: 18.0) | 116 | 25.3 (SD: 20.7) |
| Father hours in paid work (mean) | 93 | 43.5 (SD: 15.4) | 118 | 42.5 (SD: 15.3) |
| Mother cigarettes/day | 116 |  | 132 |  |
| none |  | 81.0% |  | 78.8% |
| 1-10/day |  | 8.6% |  | 11.4% |
| 11-20/day |  | 7.8% |  | 8.3% |
| 21-40/day |  | 2.6% |  | 1.5% |
| 41+/day |  | 0% |  | 0% |
| Father cigarettes/day | 110 |  | 127 |  |
| none |  | 81.8% |  | 74.8% |
| 1-10/day |  | 6.4% |  | 9.5% |
| 11-20/day |  | 9.1% |  | 11.0% |
| 21-40/day |  | 2.7% |  | 3.2% |
| 41+/day |  | 0.0% |  | 1.6% |
| Mother alcoholic drinks/wk mean (SD) | 112 | 2.4 (SD: 4.2) | 130 | 2.1 (SD: 3.5) |
| Father alcoholic drinks/wk mean (SD) | 107 | 4.4 (SD: 5.5) | 120 | 5.4 (SD: 7.6) |
|  |  |  |  |  |
| ***Pregnancy & Birth*** |  |  |  |  |
|  |  |  |  |  |
| Mother’s age at childbirth (mean, yrs) | 116 | 31.1 (SD: 4.7)* | 133 | 29.5 (SD: 5.1) |
| Father’s age at childbirth (mean, yrs) | 107 | 34.1 (SD: 5.7)* | 130 | 32.2 (SD: 5.7) |
| Planned pregnancy | 118 | 80.5% | 137 | 78.8% |
| planned natural |  | 72.0% |  | 74.5% |
| planned assisted |  | 8.5% |  | 4.4% |
| unplanned |  | 19.5% |  | 21.2% |
| Child adopted | 137 | 0% | 118 | 0% |
| Mother smoked during this pregnancy | 116 |  | 133 |  |
| Nil |  | 83.6% |  | 84.2% |
| Less than daily |  | 8.6% |  | 3.8% |
| 1-10/day |  | 3.5% |  | 8.3% |
| 11-20/day |  | 3.5% |  | 3.8% |
| 21-40/day |  | 0.9% |  | 0% |
| 41+/day |  | 0% |  | 0% |
| Father smoked during this pregnancy | 107 |  | 123 |  |
| Nil |  | 82.2% |  | 71.5% |
| Less than daily |  | 1.9% |  | 4.1% |
| 1-10/day |  | 5.6% |  | 4.9% |
| 11-20/day |  | 8.4% |  | 13.8% |
| 21-40/day |  | 1.9% |  | 4.9% |
| 41+/day |  | 0.0% |  | 0.8% |
| Mother major illness during pregnancy | 118 | 29.6% | 136 | 22.1% |
| Mother meds/supps during pregnancy |  |  |  |  |
| multivitamins | 115 | 28.7% | 123 | 21.1% |
| folate | 116 | 69.0% | 123 | 55.3% |
| calcium | 116 | 12.7% | 123 | 8.9% |
| iron | 116 | 30.2% | 123 | 39.0% |
| vitamin D | 116 | 4.3% | 123 | 2.4% |
| fish oil | 116 | 4.3% | 123 | 1.6% |
| antibiotics | 116 | 3.5% | 123 | 4.1% |
| other | 115 | 13.9% | 122 | 8.2% |
| Mother any alcohol during pregnancy | 114 | 19.3% | 134 | 17.2% |
| Mother any coffee during pregnancy | 114 | 57.0% | 132 | 54.6% |
| Child’s gestation (mean, weeks) | 99 | 39.4 (SD: 1.6) | 107 | 39.2 (SD: 2.0) |
| Child mode of delivery | 37 |  | 50 |  |
| normal vaginal |  | 64.9% |  | 62.0% |
| assisted vaginal |  | 10.8% |  | 14.0% |
| caesarian |  | 21.6% |  | 24.0% |
| other |  | 2.7% |  | 0% |
| Child birthweight (mean, g) | 105 | 3361.1 (SD: 533.8) | 114 | 3358.3 (SD: 685.0) |
| Child birthlength (mean, cm) | 92 | 50.1 (SD: 2.6) | 97 | 50.2 (SD: 4.0) |
| Child head circumference (mean, cm) | 77 | 35.0 (SD: 3.4) | 80 | 34.7 (SD: 2.7) |
| Child in multiple birth | 115 | 0.9%* | 131 | 6.1% |
| Child birth order (all live-born sibs) mean | 118 | 1.9 (SD:1.1) | 137 | 1.9 (SD: 1.0) |
|  |  |  |  |  |
| ***Early Life*** |  |  |  |  |
|  |  |  |  |  |
| Child breastfeeding |  |  |  |  |
| any | 117 | 90.6% | 136 | 82.4% |
| if any, age started (wks) (mean, SD) | 104 | 0.4 (SD 1.7) | 109 | 0.3 (SD 1.6) |
| if any, weeks breastfed (mean, SD) | 103 | 39.3 (SD 32.1) | 107 | 34.1 (SD 27.9) |
| Child formula feeding |  |  |  |  |
| age started (weeks) | 81 | 19.8 (SD 20.1) | 108 | 17.3 (SD 20.0) |
| weeks formula fed | 106 | 32.7 (SD 39.6) | 127 | 37.4 (29.9) |
| Child cow’s milk commence age (weeks) | 102 | 59.3 (SD 17.1) | 114 | 58.6 (SD 18.4) |
| Child solids commence age (weeks) | 109 | 22.4 (SD 5.4) | 119 | 25.9 (SD 21.9) |

| Mother smokes indoors near child | 109 |  | 125 |  |
| --- | --- | --- | --- | --- |
| usually |  | 0.0% |  | 1.6% |
| sometimes |  | 1.8% |  | 5.6% |
| never |  | 98.2% |  | 92.8% |
| Father smokes indoors near child | 103 |  | 123 |  |
| usually |  | 0.0% |  | 2.4% |
| sometimes |  | 1.0% |  | 5.7% |
| never |  | 99.0%* |  | 91.9% |
|  |  |  |  |  |

† Number of observations

* p < 0.05 vs older diagnosed cases by unadjusted logistic regression. See text for outcomes of adjusted analyses.

§ SEIFA = Socio-Economic Indexes for Areas, a measure of residential positional disadvantage based on the Australian Bureau of Statistics Census of Population and Housing. The higher the score, the lower the disadvantage.
